# Supplementary material for: Measuring conformational equilibria in allosteric proteins with time-resolved tmFRET
Source: bioRxiv. 2024 Jan 3:2023.10.09.561594. Originally published 2023 Oct 11. Preprint. [Version 2] doi: 10.1101/2023.10.09.561594 (PMC10592786; doi:10.1101/2023.10.09.561594)

bioRxiv preprint doi: https://doi.org/10.1101/2023.10.09.561594; this version posted January 3, 2024. The copyright holder for this preprint (which was not certified by peer review) is the author/funder, who has granted bioRxiv a license to display the preprint in perpetuity. It is made available under aCC-BY 4.0 International license.

SUPPLEMENTAL FIGURE LEGENDS

Figure S1

Gaussian model for time-resolved tmFRET. Parameters are in blue. (**A**) Plot of fluorescence lifetimes in the time domain for a single exponential donor fluorophore with time constants τ_{D_1} and τ_{D_2} in the resting and active states respectively. (**B**) Plot of the FRET efficiency (E) as a function of distance (r) showing the characteristic distance for the donor-acceptor pair (R_0). (**C**) Plot of a distribution of donor-acceptor distances ($P(r)$) with two Gaussian components with means ($\bar{r}_1$ and $\bar{r}_2$), standard deviations (σ_1 and σ_2), and fraction active (A_2). The fraction donor only (f_D) was modeled as a narrow Gaussian with a mean distance of 150 Å and a standard deviation of 0.1 Å, too far to exhibit any detectable FRET. (**D**) Plot of the out-of-phase (N_ω) and in-phase (D_ω) components of the measured, corrected, and background fluorescence response as a function of the modulation frequency (ω) where f_B is the fraction of the fluorescence intensity due to background. (**E**) Plot of the phase delay (φ_ω) and modulation ratio (m_ω) of the measured and corrected fluorescence response as a function of the modulation frequency (ω) where t_0. is the time shift of the IRF.

Figure S2

Identifiability of parameters in Gaussian model. (**A-D**) Plots of χ^2 vs Gaussian means ($\bar{r}_1$ and $\bar{r}_2$) (A) and standard deviations (σ_1 and σ_2) (B), fraction active (A_2) in 5 µM and 9.2 µM maltose (C), and fraction donor only (f_D) (D). For each plot, minimum χ^2 was determined by global fitting with the single parameter at different fixed values, while all remaining parameters varied. Results are for a representative dataset from MBP-322Acd-278C with $[Ru(bpy)_2phenM]^{2+}$. (**E,F**) χ^2 surfaces for standard deviations (σ_1 (E) and σ_2 (F)) vs. fraction donor only (f_D) showing some correlation between these parameters in the model. For each plot the standard deviation and fraction donor only were fixed at a range of values and the minimum χ^2 was determined by global fitting. Contour lines are labeled with the minimized χ^2.

Figure S3

Convergence of the Gaussian model with different initial parameter values. (**A-D**) Plots of the initial values of parameters; $\bar{r}_1$ and $\bar{r}_2$ (A), σ_1 and σ_2 (B), A_2 in 5 µM and 9.2 µM maltose (C), and A_2 in 5 µM and f_D (D); before convergence (blue circles) and after convergence (green circles) for different pairs of parameters. Initial conditions that did not converge or attain a minimum χ^2 less than 3000 are shown in red. For these calculations, the initial values for the shown parameters were varied randomly between the limits indicated by the axes, and global fitting was performed allowing all of the parameters to vary. All initial values in blue converged to nearly identical values for the parameters. Results are for a representative dataset from MBP-322Acd-278C with $[Ru(bpy)_2phenM]^{2+}$.

Figure S4

Cu^{2+} CW EPR. (**A**) X-band CW EPR spectra recorded at 112 K. Spectra are normalized by spectral intensity. Best fit simulations for each spectrum, performed in EasySpin, are shown as dashed curves. (**B**) Table of fitted magnetic parameters g and A from simulations shown in panel A.

bioRxiv preprint doi: https://doi.org/10.1101/2023.10.09.561594; this version posted January 3, 2024. The copyright holder for this preprint (which was not certified by peer review) is the author/funder, who has granted bioRxiv a license to display the preprint in perpetuity. It is made available under aCC-BY 4.0 International license.

Figure S5

RIDME zero-time artifact removal by division. 5-pulse RIDME time-traces measured at 20 K (black) contained a sharp echo-crossing artifact at $t \approx 0$ that was not removable through phase cycling. RIDME time-traces measured at 10 K (blue) also contain the zero-time artifact, but lack significant dipolar modulation, as the relaxation interval, T_R, is only ~ 4% of the Cu^{2+} T_{1e} at this temperature. Division of the 20 K data set by the 10 K data set removes the zero-time artifact, as well as much of the intermolecular background decay, but preserves the RIDME dipolar modulation (red trace). Data are shown for $[Cu(phenM)]^{2+}$ labeled MBP-295C-211C apo (A), MBP-295C-211C holo (B), MBP-322C-278C apo (C), and MBP-322C-278C holo (D).

bioRxiv preprint doi: https://doi.org/10.1101/2023.10.09.561594; this version posted January 3, 2024. The copyright holder for this preprint (which was not certified by peer review) is the author/funder, who has granted bioRxiv a license to display the preprint in perpetuity. It is made available under aCC-BY 4.0 International license.

Figure S1

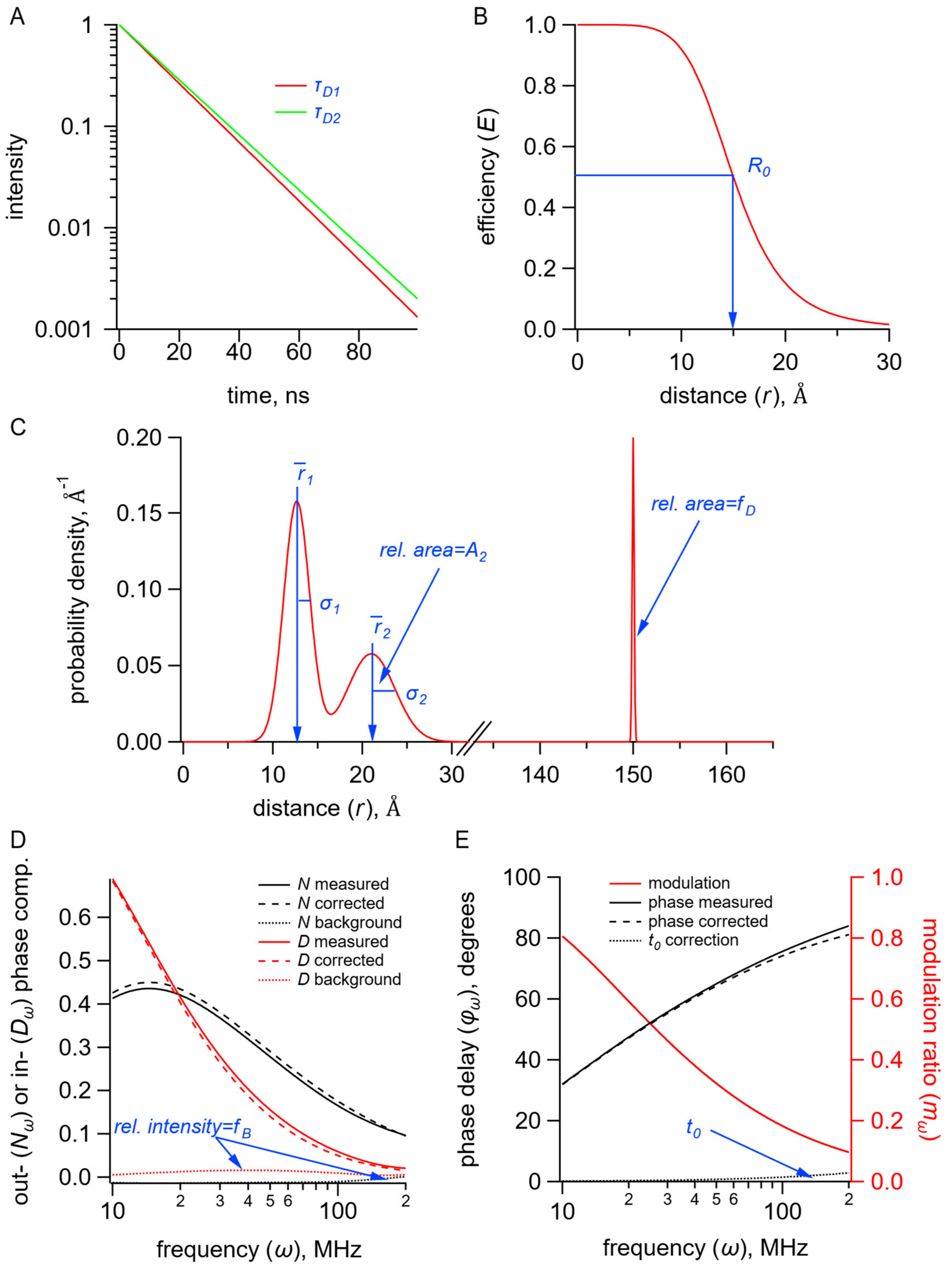

bioRxiv preprint doi: https://doi.org/10.1101/2023.10.09.561594; this version posted January 3, 2024. The copyright holder for this preprint (which was not certified by peer review) is the author/funder, who has granted bioRxiv a license to display the preprint in perpetuity. It is made available under aCC-BY 4.0 International license.

Figure S2

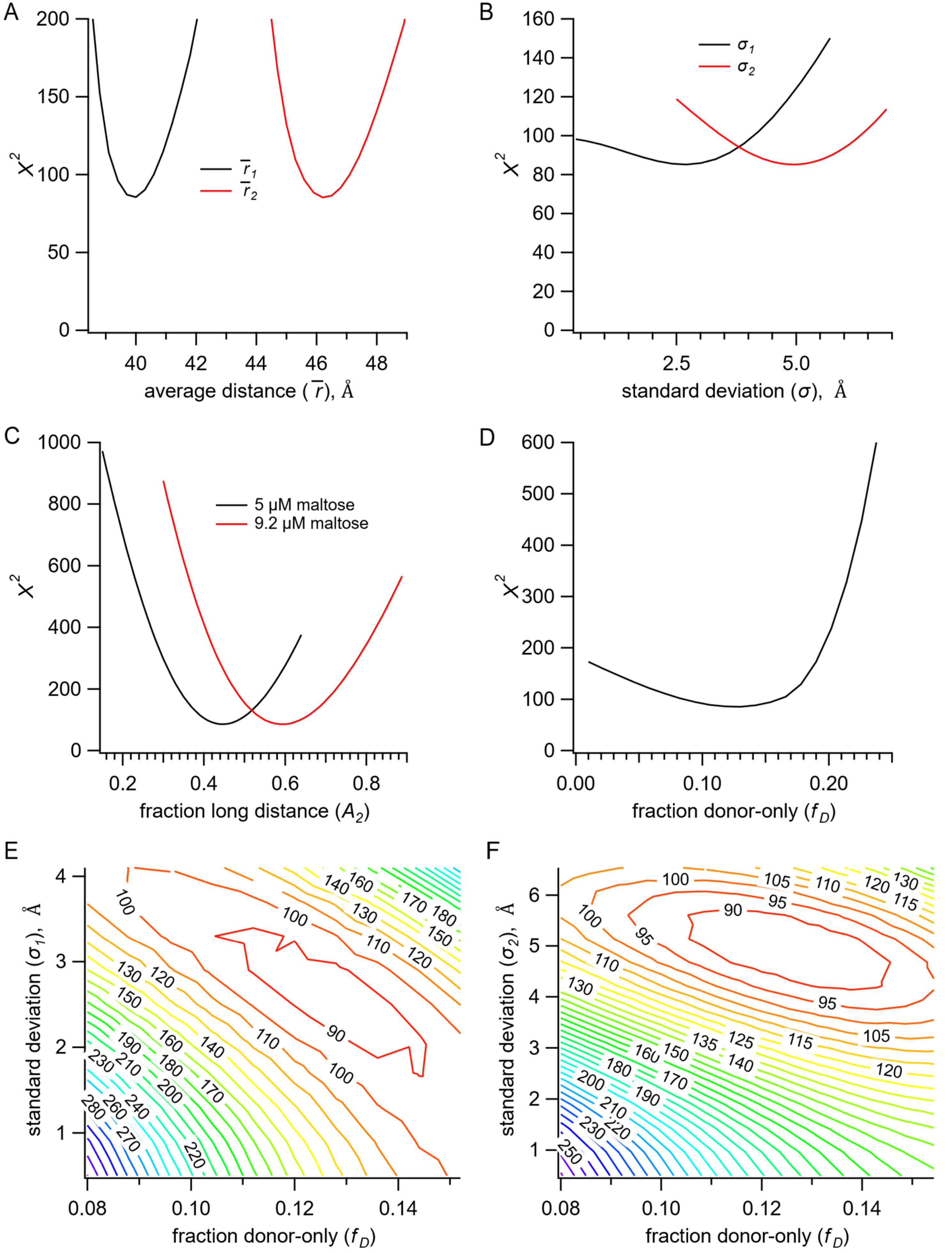

bioRxiv preprint doi: https://doi.org/10.1101/2023.10.09.561594; this version posted January 3, 2024. The copyright holder for this preprint (which was not certified by peer review) is the author/funder, who has granted bioRxiv a license to display the preprint in perpetuity. It is made available under aCC-BY 4.0 International license.

Figure S3

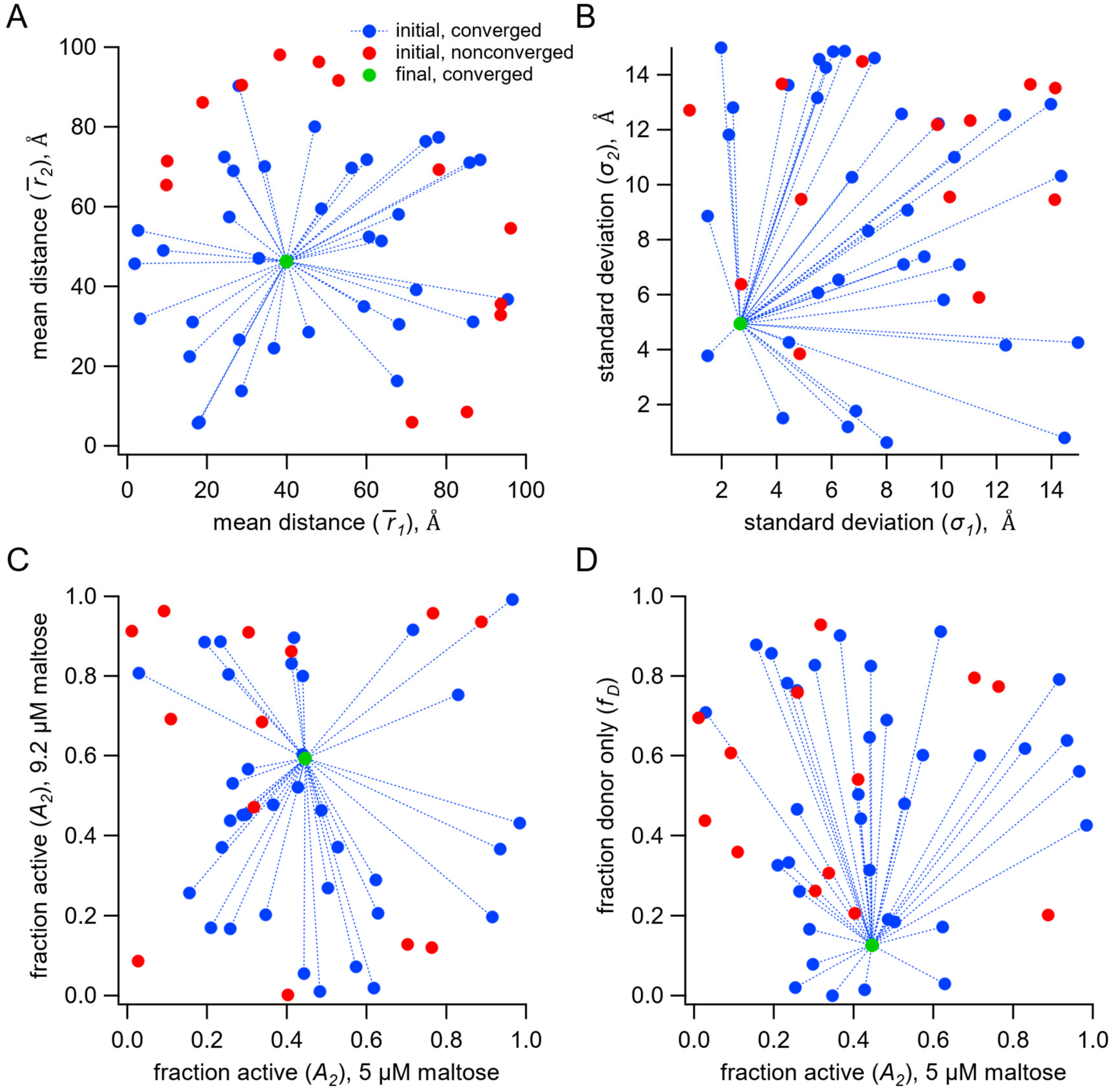

bioRxiv preprint doi: https://doi.org/10.1101/2023.10.09.561594; this version posted January 3, 2024. The copyright holder for this preprint (which was not certified by peer review) is the author/funder, who has granted bioRxiv a license to display the preprint in perpetuity. It is made available under aCC-BY 4.0 International license.

Figure S4

A

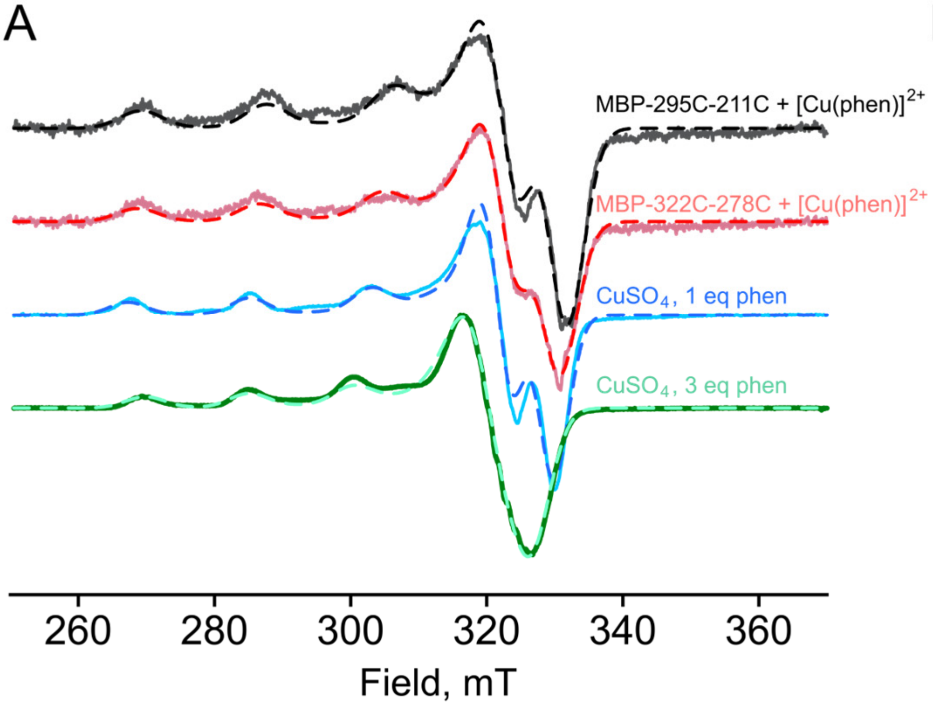

B

	g⊥	A⊥ (MHz)	g‖	A‖ (MHz)
MBP-295C-211C + $[Cu(phen)]^{2+}$	2.059	29	2.239	565
MBP-322C-278C + $[Cu(phen)]^{2+}$	2.060	21	2.249	552
Cu^{2+}:phen (1:1)	2.062	20	2.259	551
Cu^{2+}:phen (1:3)	2.070	21	2.271	473

bioRxiv preprint doi: https://doi.org/10.1101/2023.10.09.561594; this version posted January 3, 2024. The copyright holder for this preprint (which was not certified by peer review) is the author/funder, who has granted bioRxiv a license to display the preprint in perpetuity. It is made available under aCC-BY 4.0 International license.

Figure S5

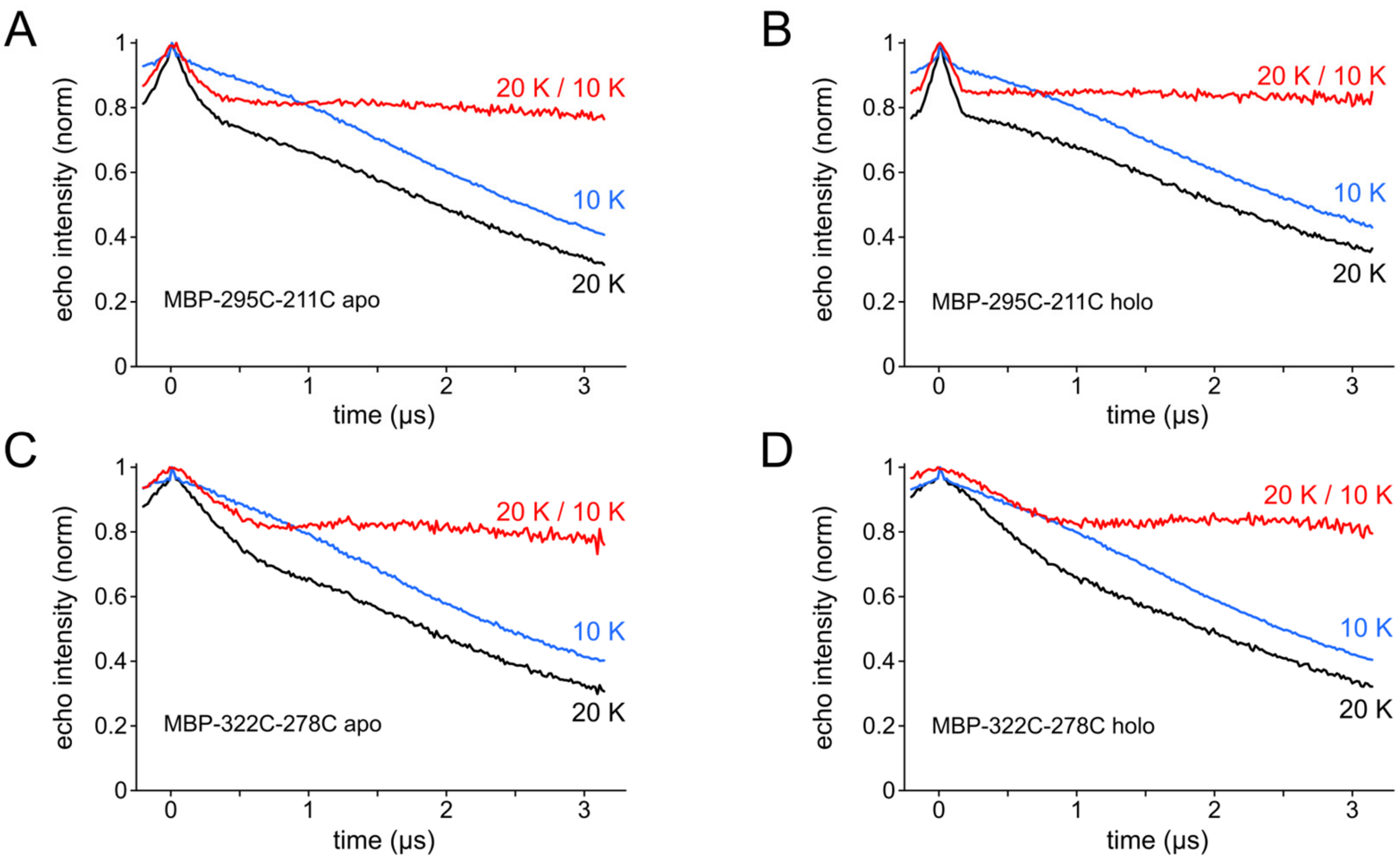

Supplement: Supplement 1 [file NIHPP2023.10.09.561594v2-supplement-1.pdf]
